# Hydrophobic insertion-based engineering of tumor cell-derived exosomes for SPECT/NIRF imaging of colon cancer

**DOI:** 10.1186/s12951-020-00746-8

**Published:** 2021-01-06

**Authors:** Boping Jing, Yongkang Gai, Ruijie Qian, Zhen Liu, Ziyang Zhu, Yu Gao, Xiaoli Lan, Rui An

**Affiliations:** 1 Department of Nuclear Medicine, Union Hospital, Tongji Medical College, Huazhong University of Science and Technology, No. 1277 Jiefang Ave, Wuhan, 430022 Hubei China; 2 Hubei Key Laboratory of Molecular Imaging, Wuhan, 430022 China

**Keywords:** Tumor cell-derived exosomes, SPECT/NIRF imaging, Colon cancer, Hydrophobic insertion

## Abstract

**Background:**

Tumor cell-derived exosomes (TEx) have emerged as promising nanocarriers for drug delivery. Noninvasive multimodality imaging for tracing the in vivo trafficking of TEx may accelerate their clinical translation. In this study, we developed a TEx-based nanoprobe via hydrophobic insertion mechanism and evaluated its performance in dual single-photon emission computed tomography (SPECT) and near-infrared fluorescence (NIRF) imaging of colon cancer.

**Results:**

TEx were successfully isolated from HCT116 supernatants, and their membrane vesicle structure was confirmed by TEM. The average hydrodynamic diameter and zeta potential of TEx were 110.87 ± 4.61 nm and –9.20 ± 0.41 mV, respectively. Confocal microscopy and flow cytometry findings confirmed the high tumor binding ability of TEx. The uptake rate of ^99m^Tc-TEx-Cy7 by HCT116 cells increased over time, reaching 14.07 ± 1.31% at 6 h of co-incubation. NIRF and SPECT imaging indicated that the most appropriate imaging time was 18 h after the injection of ^99m^Tc-TEx-Cy7 when the tumor uptake (1.46% ± 0.06% ID/g) and tumor-to-muscle ratio (8.22 ± 0.65) peaked. Compared with radiolabeled adipose stem cell derived exosomes (^99m^Tc-AEx-Cy7), ^99m^Tc-TEx-Cy7 exhibited a significantly higher tumor accumulation in tumor-bearing mice.

**Conclusion:**

Hydrophobic insertion-based engineering of TEx may represent a promising approach to develop and label exosomes for use as nanoprobes in dual SPECT/NIRF imaging. Our findings confirmed that TEx has a higher tumor-targeting ability than AEx and highlight the potential usefulness of exosomes in biomedical applications.
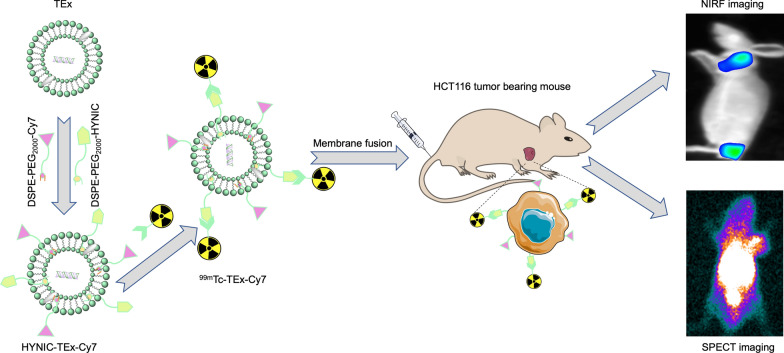

## Background

Colon cancer is an extremely complex and multifactorial disease, causing millions of deaths every year [1]. Although colonoscopy is widely used to diagnose colon cancer, it offers limited sensitivity and specificity for early-stage disease. Molecular imaging is a noninvasive or minimally invasive alternative, providing a detailed insight into the physiological and pathological processes of the human body; hence, molecular imaging is more likely to diagnose cancer at an early stage [2, 3]. Each imaging technique offers different spatial resolution, sensitivity, depth of tissue penetration, cost, and time resolution. Multimodality imaging combines the advantages of different imaging technologies, providing comprehensive, three-dimensional information, as well as a more accurate spatial positioning and molecular information ideal for the detection of small lesions [4]. Hence, the development of multimodality molecular imaging agents has gained increasing attention over the last years. The agent requires a suitable carrier with certain intrinsic properties, such as large carrying capacity and facile surface modification. Synthetic nanoparticles, including liposomes, metal nanoparticles, and magnetic nanoparticles, have a broad clinical application in multimodality imaging [5–7]. However, most of them are artificial drug carriers possessing potential toxicity, immunogenicity, and inability to penetrate most organs [8].

Exosomes have emerged as promising natural nanocarriers due to their nontoxicity and biocompatibility [9, 10]. They are extracellular vesicles of endosomal origin secreted by almost all types of cells [11]. In the past decade, exosomes have emerged as novel nanocarriers in drug delivery systems owing to their suitable particle size (30–150 nm), high stability, and large carrying capacity [12]. Furthermore, exosome-based drug delivery harnesses endogenous mechanisms for uptake, intracellular trafficking, and subsequent delivery of the cargo [10]. To date, different types of exosomes have been developed to deliver drugs to tumors; tumor cell-derived exosomes (TEx), adipose stem cell-derived exosomes (AEx), and epidermal cell-derived exosomes are among the most promising ones [13–17]. Exosomes from different cells have differential properties, and TEx have inherent tumor-targeting capabilities [18]. Additionally, various biomedical imaging modalities have been modified to trace exosomes. These modalities include magnetic resonance (MR), single-photon emission computed tomography (SPECT), positron emission tomography (PET), and optical imaging [19–24]; among these, SPECT and optical imaging are currently the most commonly used due to their low cost and wide availability. SPECT can be used to image the whole body and offers excellent penetration; however, SPECT imaging is limited by the relatively long acquisition time, short imaging time window, and low spatial resolution. Near-infrared fluorescence (NIRF, 650–1000 nm) imaging offers real-time and high-resolution tissue structure information [25, 26], although tissue penetration is limited. Multimodality SPECT and NIRF imaging can provide complementary insight into disease progression and real-time tumor delineation [27]. To the best of our knowledge, there are currently no exosome-based nanoprobes for multimodality SPECT and NIRF imaging.

To visualize the exosomes, functional molecules should be introduced on exosomes’ surfaces to modify exosomes for multimodal imaging. However, it remains a challenge to modify different functional groups on the surface of exosomes due to the small size and complex surface chemistry of exosomes [28]. Here, we propose a hydrophobic insertion strategy to modify TEx with DSPE-PEG_2000_-Cy7 and DSPE-PEG_2000_-HYNIC. The modified TEx (HYNIC-TEx-Cy7) was labeled with ^99m^Tc, allowing for SPECT and NIRF imaging of tumor-bearing nude mice in vivo. AEx labeled with ^99m^Tc and Cy7 (^99m^Tc-AEx-Cy7) were used for comparison.

## Results

### TEx and AEx isolation and characterization

**Fig. 1:**
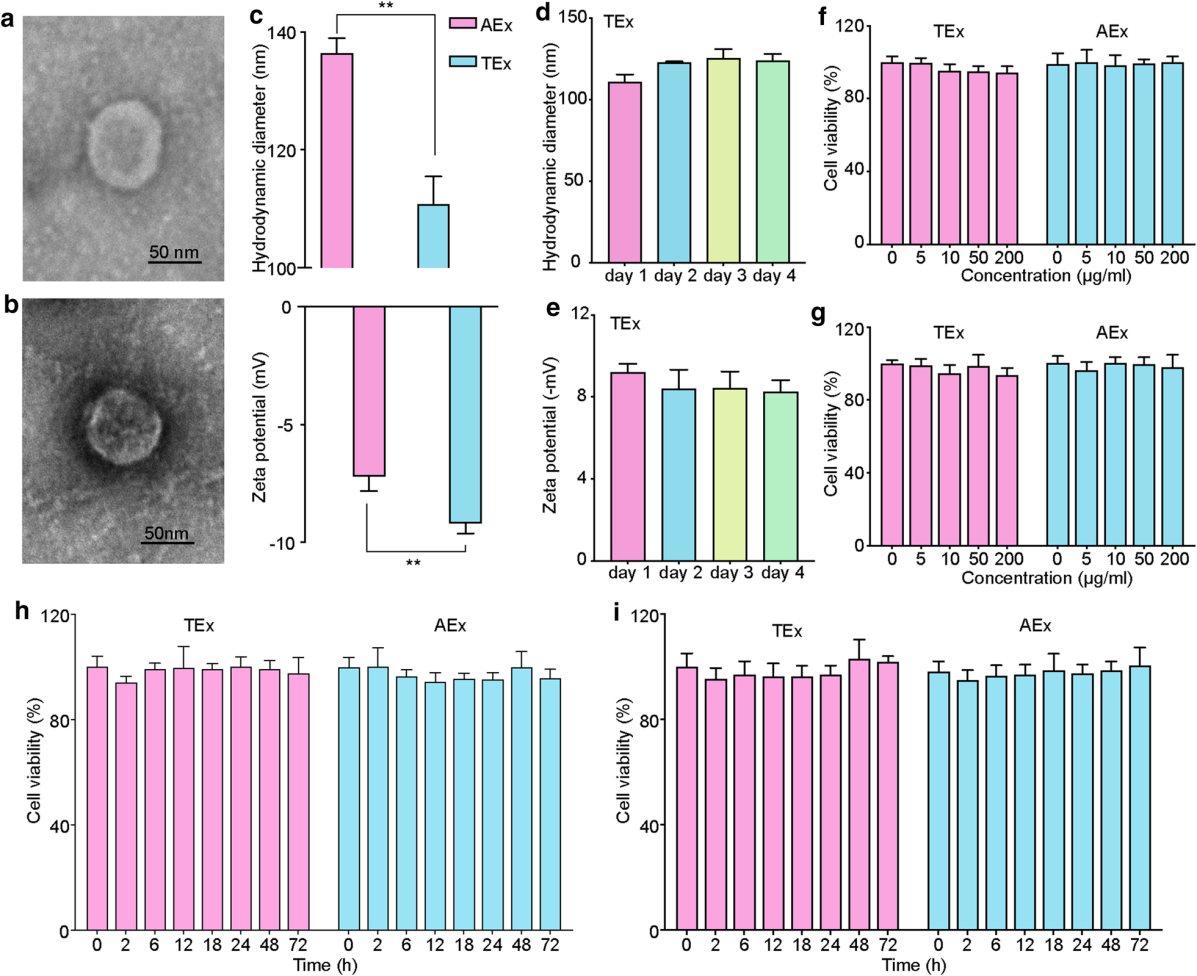
The identification of TEx/AEx and the stability of TEx. **a** Transmission electron microscopy (TEM) images of exosomes isolated from HCT116 cells (TEx). **b** TEM images of exosomes isolated from adipose stem cells (AEx). **c** The average hydrodynamic diameters and zeta potential of TEx/AEx. **d** The average hydrodynamic diameters of TEx within 4 days. **e** The zeta potential of TEx within 4 days. **f**, **g** The HCT116 cell and adipose stem cell viability after 24 h incubation with TEx and AEx at different concentrations. **h**, **i** The HCT116 cell and adipose stem cell viability after incubation with TEx and AEx at different time points. Bars represent means ± SD (n = 3)

TEx and AEx were successfully isolated from the supernatant of tumor cells and adipose stem cells (ASCs), respectively. Expectedly, TEx and AEx appeared as membrane vesicles under a TEM (Fig. 1a, b). The average hydrodynamic diameters of TEx and AEx were 110.87 ± 4.61 nm and 136.47 ± 2.50 nm, respectively. The zeta potentials of TEx and AEx were –9.20 ± 0.41 mV and –7.22 ± 0.60 mV, respectively (Fig. 1c). The average hydrodynamic diameter and zeta potential of TEx did not change significantly for up to 4 days, indicated the excellent stability (Fig. 1d, e). A CCK-8 assay was performed to evaluate the cell cytotoxicity of exosomes to HCT116 colon cancer cells. HCT116 cancer cells or adipose stem cell were co-incubated with TEx and AEx at various concentrations (up to 200 μg/mL) and different time periods (up to 72 h). The results showed that the survival rate of cells in each group was greater than 90% (Fig. 1f–i). TEx and AEx had no obvious toxicity to HCT116 colon cancer cell and adipose stem cell.

### In vitro tumor cell binding

**Fig. 2:**
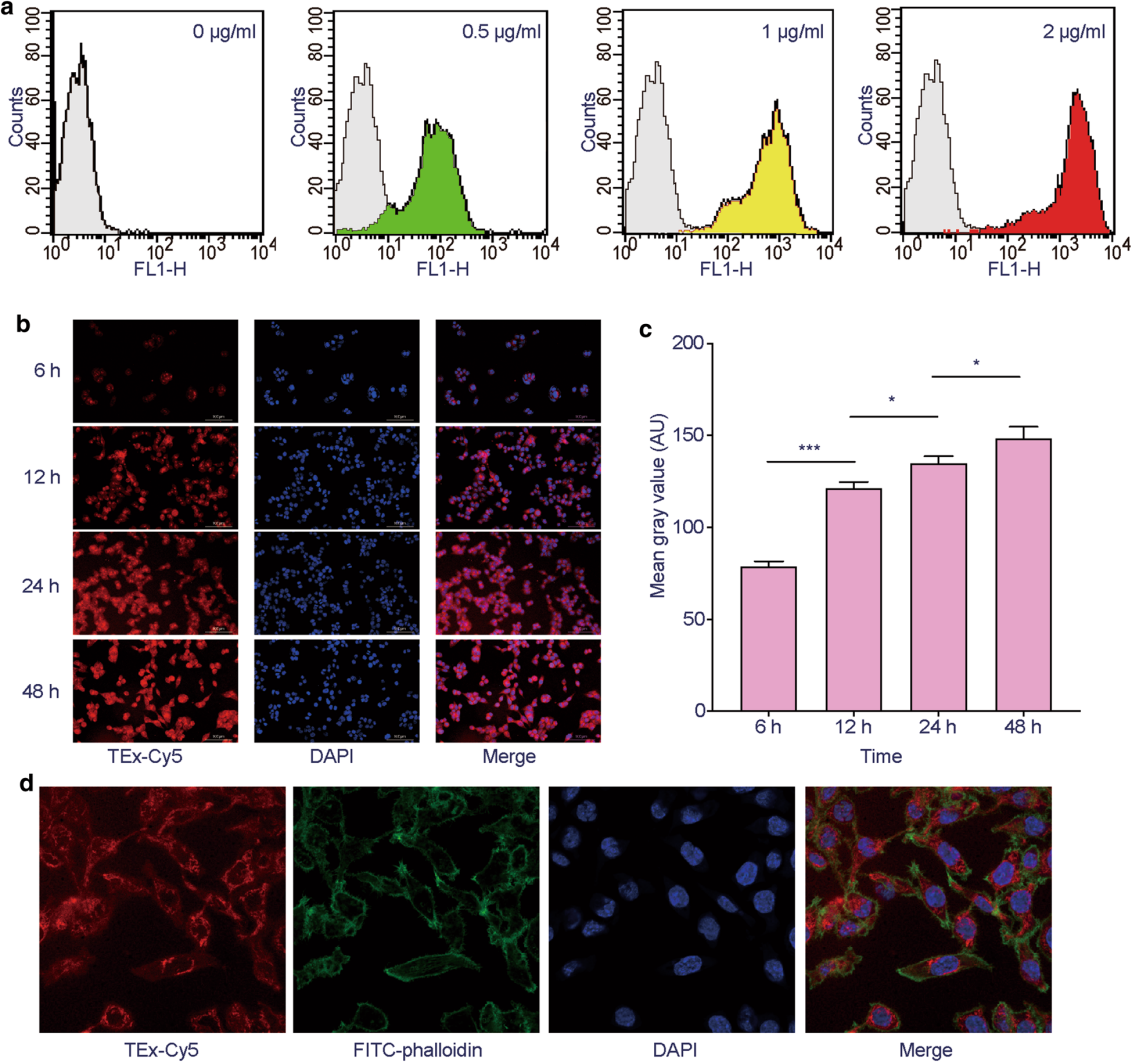
Tumor-binding ability of TEx. **a** Flow cytometry analysis of HCT116 cells incubated with the increasing concentrations of Cy5-labeled TEx. **b** Fluorescence images of HCT116 cells after incubating with Cy5-labeled TEx for different time periods (200 ×). **c** Corresponding quantification of the fluorescent intensity. (E) Confocal microscopy images of HCT116 cells incubated with Cy5-labeled TEx (600 ×)

After a 12-h incubation of FITC-TEx with HCT116 cell, tumor cells were analyzed by flow cytometry. The fluorescence intensity of tumor cells was increased with increasing concentrations of FITC-TEx, reaching a maximum when cells were co-cultured with 20 μg/mL of FITC-TEx (Fig. 2a). As exhibited in fluorescence images, the uptake of Cy5-labeled TEx in HCT116 cell was increased over time, but the uptake did not change significantly after 12 h (Fig. 2b). The quantification of the fluorescent intensity was consistent with images (Fig. 2c). Confocal microscopy of HCT116 cells incubated with Cy5-labeled TEx revealed strong fluorescence signals in the cell membrane and cytoplasm (Fig. 2d).

### Exosome radiolabeling, purification, and in vitro cellular uptake

**Fig. 3:**
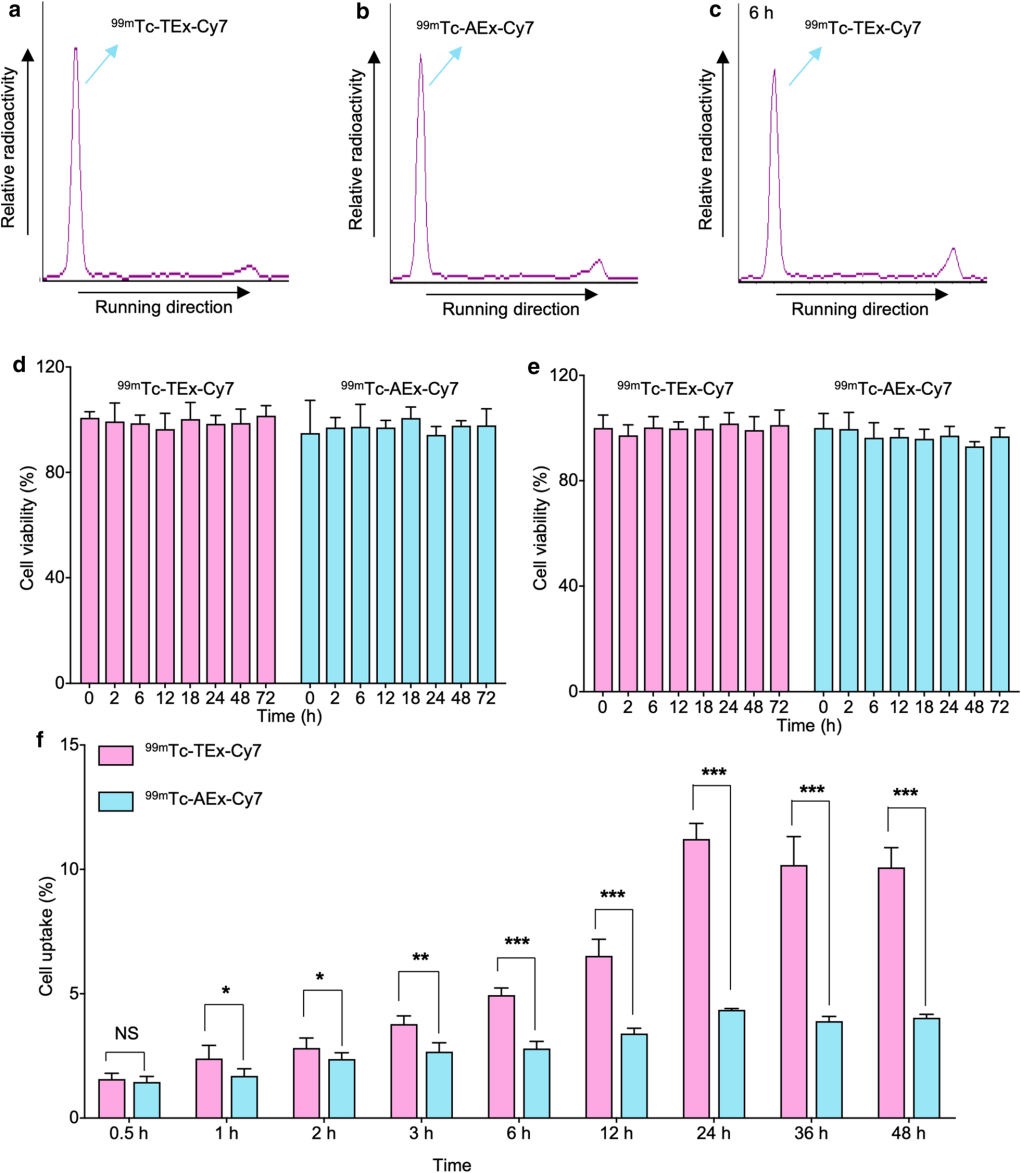
The representative radiochemical purity and cell uptakes of ^99m^Tc-TEx-Cy7/ ^99m^Tc-AEx-Cy7. **a** The representative radiochemical purity of ^99m^Tc-TEx-Cy7. **b** The representative radiochemical purity of ^99m^Tc-AEx-Cy7 **c** The serum stability of ^99m^Tc-TEx-Cy7 at 6 h. **d**, **e** The HCT116 cell and adipose stem cell viability after incubation with ^99m^Tc-TEx-Cy7 and ^99m^Tc-AEx-Cy7 at different time points. **f** Uptakes of ^99m^Tc-TEx-Cy7 and ^99m^Tc-AEx-Cy7 in HCT116 tumor cells at the indicated time points. Bars represent means ± SD (n = 3)

The radiochemical purities of ^99m^Tc-TEx-Cy7 and ^99m^Tc-AEx-Cy7 were 94.77% ± 1.87% and 91.12% ± 2.72%, respectively (Fig. 3a, b). The proportion of intact tracer (^99m^Tc-TEx-Cy7) was 81.03% ± 0.98% after 6-h incubation in FBS at 37℃ (Fig. 3c). FT-IR (Fourier transform infrared) spectrum displayed a significant change between TEx and ^99m^Tc-TEx-Cy7 (Additional file 1: Figure S1) indicating the successful insertion of DSPE-PEG materials. HCT116 cancer cells or adipose stem cells were co-incubated with ^99m^Tc-TEx-Cy7 and ^99m^Tc-AEx-Cy7 TEx at different time periods (up to 72 h). The results showed that the survival rate of cells in each group was greater than 90% (Fig. 3d, e). ^99m^Tc-TEx-Cy7 and ^99m^Tc-AEx-Cy7 had no obvious toxicity to HCT116 cancer cells and ASCs. The uptake rate of ^99m^Tc-TEx-Cy7 in HCT116 cells increased over time and peaked at 24 h, then decreased gradually. Importantly, the uptake rates of ^99m^Tc-TEx-Cy7 were significantly higher than those of ^99m^Tc-AEx-Cy7 at all investigated time points (Fig. 3f).

### In vivo NIRF imaging

**Fig. 4:**
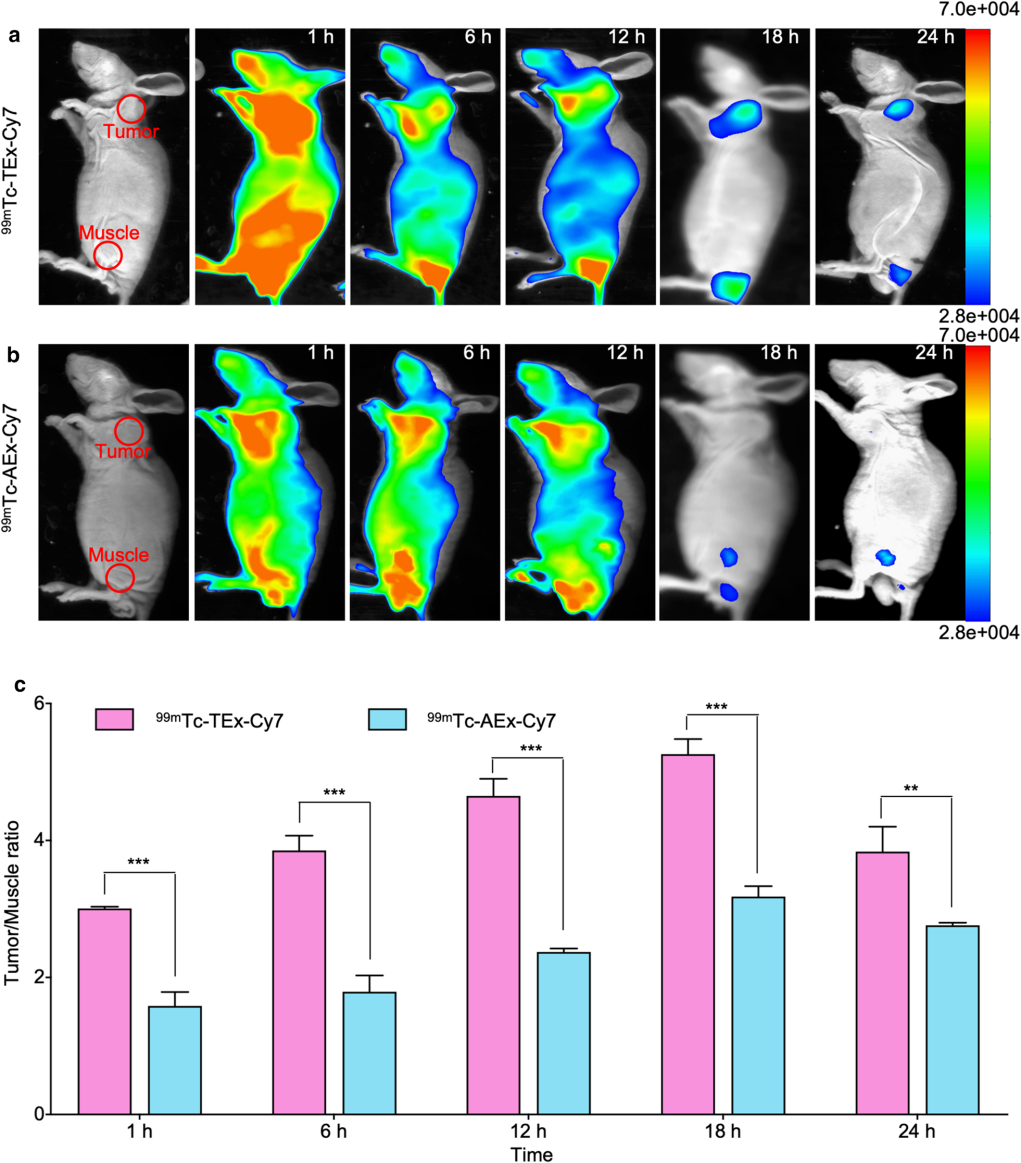
NIRF imaging of tumor-bearing nude mice (n = 3). **a** The NIRF images of tumor-bearing nude mice at different times (1, 6, 12, 18 and 24 h) after the injection of ^99m^Tc-TEx-Cy7. **b** The NIRF images of tumor-bearing nude mice at different times (1, 6, 12, 18 and 24 h) after the injection of ^99m^Tc-AEx-Cy7. **c** Tumor/Muscle ratios at different times after the injection and their comparations (n = 3, **P < 0.01; ***P < 0.001)

NIRF imaging was performed 1, 6, 12, 18, and 24 h after the administration of ^99m^Tc-TEx-Cy7 and ^99m^Tc-AEx-Cy7, and the changes in the biodistribution of the multimodality nanoprobes were observed over time. NIRF imaging revealed that ^99m^Tc-TEx-Cy7 (Fig. 4a) were taken up by tumor cells at a higher rate than ^99m^Tc-AEx-Cy7 (Fig. 4b). The quantification of the fluorescent intensity was consistent with NIRF images (Fig. 4c). The most appropriate time for NIRF imaging was 18 h after the injection of ^99m^Tc-TEx-Cy7.

### In vivo SPECT imaging

**Fig. 5:**
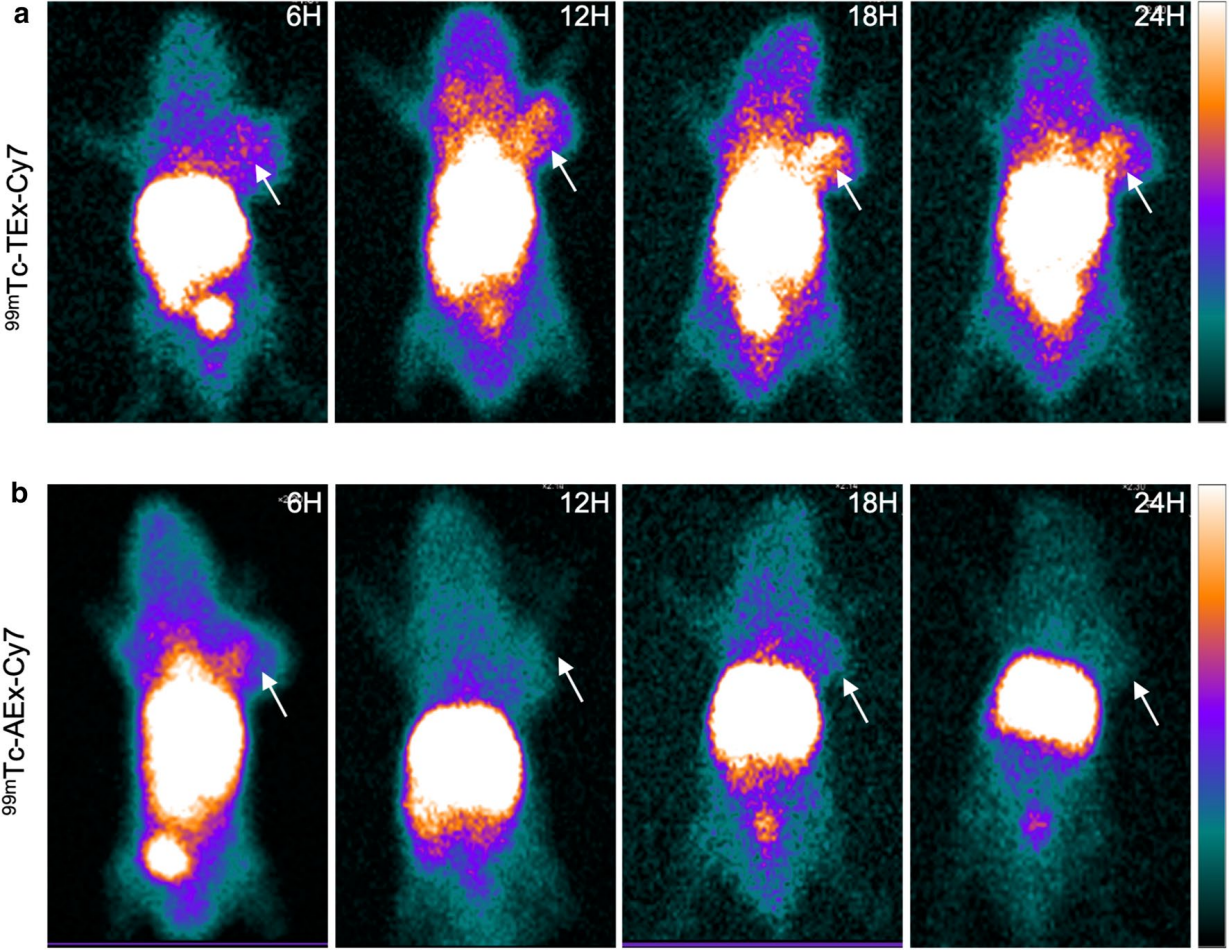
SPECT imaging of tumor-bearing nude mice. **a** The SPECT images of tumor-bearing nude mice at different times (6, 12, 18 and 24 h) after the injection of ^99m^Tc-TEx-Cy7. **b** As a control group, the SPECT images of tumor-bearing nude mice at different times (6, 12, 18 and 24 h) after the injection of ^99m^Tc-AEx-Cy7. The white arrow points to the tumor site

SPECT imaging was performed 6, 12, 18, and 24 h after the administration of ^99m^Tc-TEx-Cy7 and ^99m^Tc-AEx-Cy7. Tumor-bearing mice exhibited an accumulation of the multimodality nanoprobe in the abdominal cavity. Compared with the ^99m^Tc-AEx-Cy7 group, the ^99m^Tc-TEx-Cy7 group exhibited a higher tumor uptake of the tracer (Fig. 5a, b). The most appropriate time for SPECT imaging was 18 h after the injection of ^99m^Tc-TEx-Cy7.

### Biodistribution

**Fig. 6:**
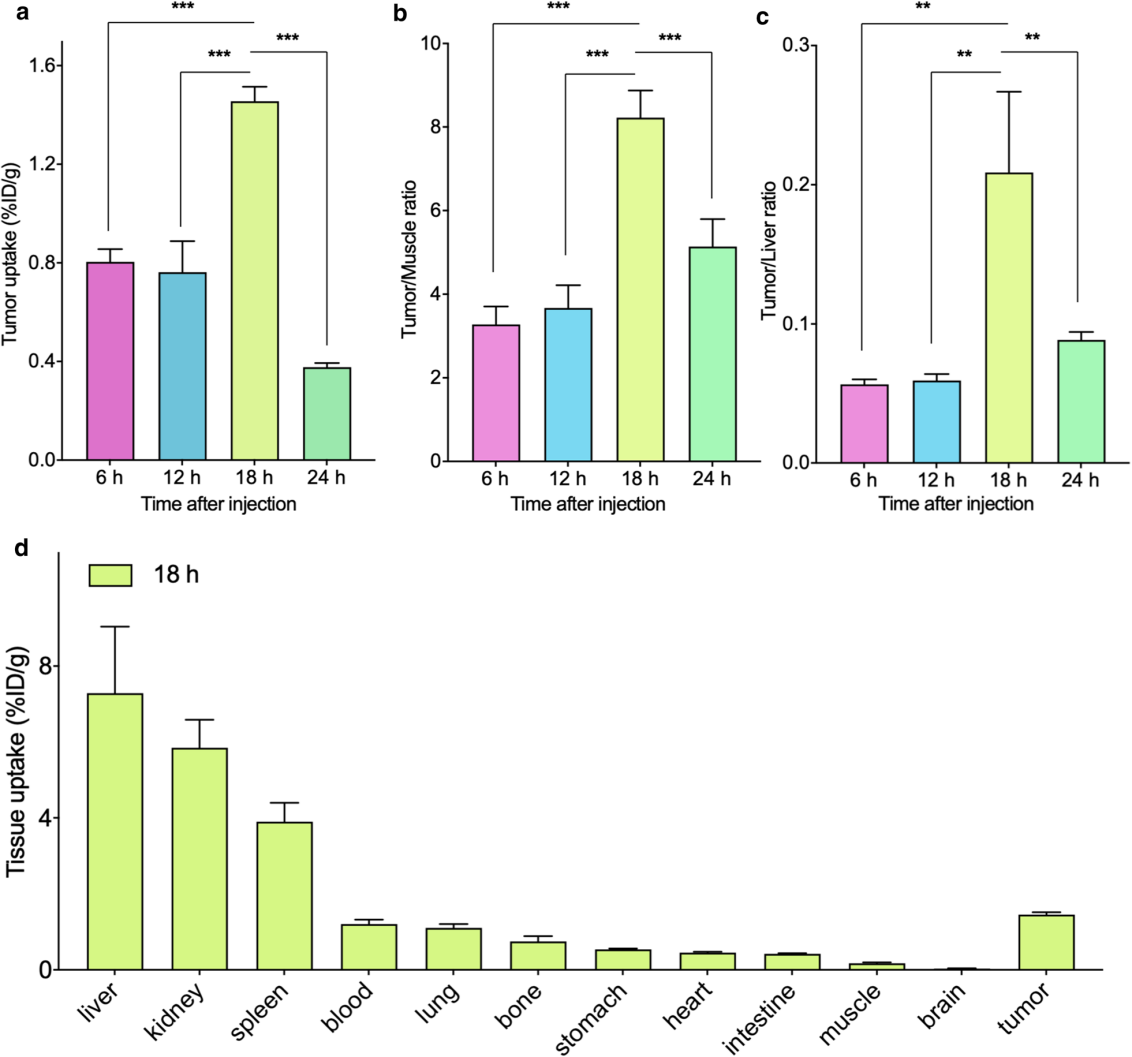
Biodistribution analysis of different time points (6, 12, 18 and 24 h) after the injection ^99m^Tc-TEx-Cy7. **a** Tumor uptakes of different time points. **b**, **c** Tumor/Muscle ratios, Tumor/Liver ratios of different time points. **d** Tissues uptakes of HCT116 tumor-bearing mice at 18 h after injection. All bars represent means ± SD (n = 3). *P < 0.05; **P < 0.01; ***P < 0.001

The findings of biodistribution analyses were consistent with those of SPECT imaging (Table 1). Tumor uptake of ^99m^Tc-TEx-Cy7 reached a maximum of 1.46% ± 0.06% ID/g at 18 h after injection (Fig. 6a). Notably, the tumor-to-muscle (T/M) and tumor-to-liver (T/L) ratios reached a maximum 18 h after injection of ^99m^Tc-TEx-Cy7 (Fig. 6b, c). As shown in Fig. 6d, the liver of tumor-bearing mice exhibited the highest radioactive uptake (14.29% ± 1.73% ID/g at 6 h; 12.78% ± 1.45% ID/g at 12 h; 7.28% ± 1.76% ID/g at 18 h; and 4.27% ± 0.31% ID/g at 24 h), followed by the kidneys (7.35% ± 0.87% ID/g at 6 h; 6.02% ± 0.30% ID/g at 12 h; 5.85% ± 0.74% ID/g at 18 h; and 2.15% ± 0.72% ID/g at 24 h).

**Table 1:** The biodistribution of different time points (6, 12, 18 and 24 h) after the injection of ^99m^Tc-TEx-Cy7. Data are expressed as mean ± standard deviation (n = 3)

Tissues	^99m^Tc-TEx-Cy7			
	6 h	12 h	18 h	24 h
Blood	1.85 ± 0.31	2.30 ± 0.37	1.21 ± 0.11	0.40 ± 0.03
Brain	0.07 ± 0.01	0.05 ± 0.01	0.04 ± 0.00	0.02 ± 0.01
Heart	0.49 ± 0.05	0.41 ± 0.04	0.46 ± 0.02	0.18 ± 0.05
Lung	2.23 ± 0.60	1.13 ± 0.12	1.11 ± 0.10	0.73 ± 0.41
Liver	14.29 ± 1.73	12.78 ± 1.45	7.28 ± 1.76	4.27 ± 0.31
Spleen	5.11 ± 0.94	3.32 ± 0.51	3.90 ± 0.50	1.33 ± 0.17
Kidney	7.35 ± 0.87	6.02 ± 0.30	5.85 ± 0.74	2.15 ± 0.72
Stomach	4.60 ± 1.54	0.86 ± 0.35	0.57 ± 0.06	0.24 ± 0.14
Intestine	0.77 ± 0.24	0.56 ± 0.08	0.42 ± 0.02	0.23 ± 0.05
Bone	1.29 ± 0.33	0.94 ± 0.13	0.75 ± 0.14	0.40 ± 0.13
Muscle	0.25 ± 0.05	0.21 ± 0.01	0.18 ± 0.02	0.11 ± 0.06
Tumor	0.80 ± 0.05	0.76 ± 0.13	1.46 ± 0.06	0.38 ± 0.02

### In vivo toxicity studies

**Fig. 7:**
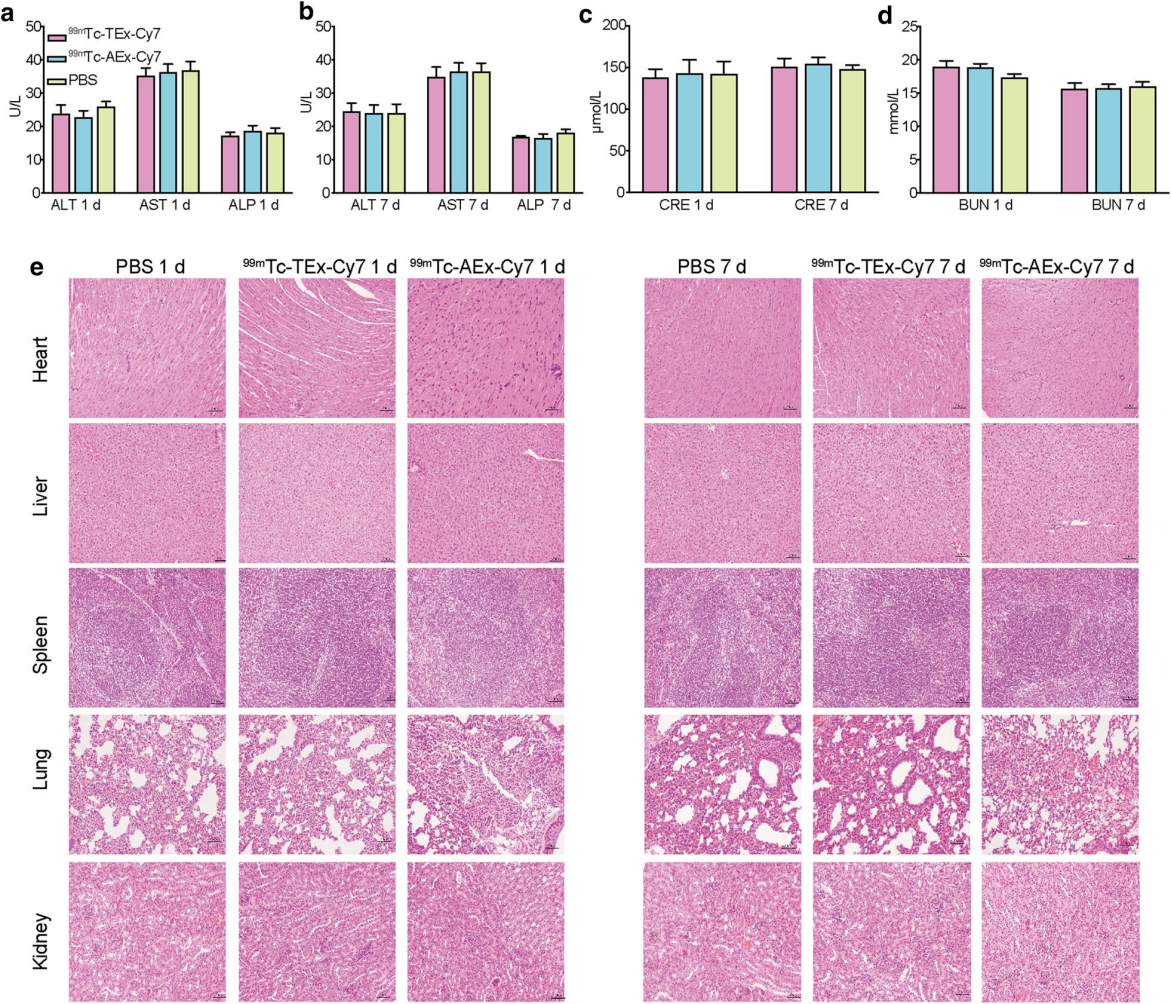
In vivo toxicity evaluation by blood test and histology analysis. **a**–**d** Liver function makers (ALT, AST and ALP) and kidney function markers (BUN and CRE) after i.v. injection with PBS or ^99m^Tc-TEx-Cy7 or ^99m^Tc-AEx-Cy7 over 1 d and 7 d. **e** Representative H&E staining images of major organs from the euthanized mice. Bar = 50 μm. All error bars represent standard deviations (n = 5)

BALB/c mice (n = 5) received an i.v. injection of 200 μL of PBS, or PBS containing ^99m^Tc-TEx-Cy7 or ^99m^Tc-AEx-Cy7 to evaluate the in vivo potential toxicity. No significant hepatic or renal toxicity was observed from the indicating normal values of liver and kidney function markers, including ALT, AST, ALP, BUN and CRE (Fig. 7a–d). Also, we did not observe significantly evidence of major organ damage from the H&E stained sections (Fig. 7e).

## Discussion

In this study, we employed a hydrophobic insertion method to label TEx with a radionuclide (^99m^Tc) and NIRF dye (Cy7) and develop a multimodality imaging nanoprobe (^99m^Tc-TEx-Cy7) targeting colon cancer. Our in vivo and in vitro findings indicated the high affinity of the probe for tumor cells. To the best of our knowledge, this is the first study to use TEx as a nanocarrier for multimodal SPECT and NIRF imaging.

TEx offer exhibited several favorable characteristics as natural nanocarriers, such as suitable nanoparticle sizes (110.87 ± 4.61 nm), negative zeta potential (–9.20 ± 0.41 mV), no obvious toxicity and high biocompatibility, making them ideal for various biomedical applications. As revealed by the results of DLS, the average hydrodynamic diameter and zeta potential remained similar for up to 4 days, indicating excellent stability. Flow cytometry analysis, fluorescence imaging and confocal imaging revealed that the nanoprobe had good tumor cell binding ability and that a major porpertion nanoprobes were internalized by tumor cells. The nanoprobe exhibited no obvious cytotoxicity, as shown by in vitro and in vivo toxicity studies.

Several modification strategies have been proposed to modify exosomes; these approaches include antigen–antibody binding, genetic engineering, loading, and hydrophobic interaction [29]. Hydrophobic interactions are ideal for incorporating various functional groups on the surface of exosomes, offering rapid reaction, simplicity, low cost, and high yield. Additionally, hydrophobic insertion can be used to engineer virtually all exosome types without affecting the morphology and biological properties of exosomes [28]. Hydrophobic and amphiphilic materials can penetrate lipid bilayers, offering an ideal platform for membrane modification. The lipid analog DSPE-PEG is widely used as a modification material to insert functional molecules on the surface of exosomes based on hydrophobic interactions with exosome membrane lipids [28]. In addition, the use of PEG can endow exosomes with the so-called “stealth” properties to reduce the protein adsorption [30, 31]. Taking account of the limitations of the device channel, TEx were modified with DSPE-PEG_2000_-FITC (TEx-FITC) and subsequently used for in vitro tumor cell uptake analysis. We found that the fluorescence signals of tumor cells were elevated with increasing concentrations of TEx-FITC. TEx were also modified with DSPE-PEG_2000_-Cy5 (TEx-Cy5) and co-incubated with tumor cells; confocal imaging revealed strong fluorescence signals in tumor cell membranes and the cytoplasm. These findings suggest the successful modification of TEx. HYNIC and Cy7 were also inserted into the surface of TEx using the same method.

The use of ^99m^Tc-HMPAO and ^99m^Tc-tricarbonyl to label exosomes has been previously reported [19, 32]. However, labeling with ^99m^Tc-HMPAO and ^99m^Tc-tricarbonyl requires is elaborate, requiring expensive and complex radioactive precursors. In this study, we used a simple labeling approach that provided radiochemical purities of > 85% for both ^99m^Tc-TEx-Cy7 and ^99m^Tc-AEx-Cy7. The radiolabeled exosomes were stable, with more than 80% remaining intact after incubation in FBS for 6 h at 37 °C.

SPECT imaging provides excellent penetration and sensitivity, whereas NIRF imaging offers high temporal resolution, spatial resolution, and real-time tumor delineation. In this study, the nanoprobe’s in vivo biodistribution was assessed using SPECT imaging, and the tumor boundaries were identified using NIRF imaging. Multimodality imaging with SPECT and NIRF can combine the advantages of SPECT and NIRF. The most appropriate SPECT and NIRF imaging time was determined to be 18 h after the injection of the nanoprobe, as a maximum tumor uptake (1.46 ± 0.06% ID/g) and tumor-to-muscle ratio (8.22 ± 0.65) was observed 18 h after injection of ^99m^Tc-TEx-Cy7.

Exosomes derived from tumor cells inherently possess a high tumor-targeting ability. Our in vivo and in vitro analyses demonstrated that ^99m^Tc-TEx-Cy7 had a high affinity for tumor cells. TEx and AEx exhibited similar average hydrodynamic diameters and zeta potentials. Nevertheless, compared with ^99m^Tc-AEx-Cy7, ^99m^Tc-TEx-Cy7 showed higher tumor cell uptake at all the time points tested and more profound tumor accumulation in tumor-bearing mice. These results suggest that TEx offer better tumor-targeting ability than AEx.

There are several limitations to this study. TEx accumulation was observed in the liver, spleen, and kidneys, impacting the quality of imaging. A pre-targeting approach for nuclear imaging could be employed to reduce the uptake in the liver and spleen. Furthermore, the production and isolation of exosomes still remain challenges. New methods for isolation of Ex are research hotspot, such as microfluidic methods.

## Conclusions

In this study, a novel exosomes-based nanoprobe was successfully engineered for multimodal SPECT/NIRF imaging of colon cancer. The use of hydrophobic interactions provides possibility for engineering exosomes-based multimodal imaging agents. Our data verified that DSPE-PEG_2000_ functionalized groups can be inserted on the surface of exosomes using this approach. This research also proved that exosomes from tumor cells are potential high-quality nanocarriers for multimodal imaging and have broad application prospects.

## Methods

### Cell culture

The study protocols were approved by the Ethics Committee at the Tongji Medical College of Huazhong University of Science and Technology. Human adipose stem cells were isolated from subcutaneous fat. The human colon cancer cell line HCT116 was maintained in our laboratory (Hubei Province Key Laboratory of Molecular Imaging) in RPMI-1640 medium (Gibco, USA) supplemented with 10% fetal bovine serum (FBS; Gibco, USA). The cells cultured at 37 °C in a humidified atmosphere containing 5% CO2.

### Isolation of TEx and AEx

Exosomes from serum-free culture supernatants of HCT116 cells and ASCs were obtained by differential ultracentrifugation. Dead cells and cell fragments were removed by centrifugation at 3000×*g* for 30 min. The supernatants were centrifuged at 13,000×*g* for 70 min to remove microvesicles. Subsequently, the supernatants were concentrated using an Amicon®Ultra-15 Centrifugal Filter Devices (100 kDa molecular weight, Millipore, USA). The supernatants were centrifuged at 120,000×*g* for 70 min and passed through a 0.22 μm filter to obtain TEx and AEx.

### TEx and AEx characterization

For transmission electron microscopy (TEM), TEx and AEx were resuspended at 1.0 mg/mL in phosphate buffer saline (PBS; Gibco, USA), placed on 200-mesh carbon-coated copper grids for 2 min, and subjected to negative staining using phosphotungstic acid. The hydrodynamic diameters and zeta potential values were identified by dynamic light scattering (DLS; Malvern Instruments Ltd., Worcestershire, UK). The changes in hydrodynamic diameters were monitored for 4 days by DLS to test the stability of TEx in vitro.

### Cell cytotoxicity assay

The HCT116 colon cancer cells or ASCs were seeded in 96-well plates at a density of 1 × 10^4^ cells per well and cultured for 12 h. TEx, AEx, ^99m^Tc-TEx-Cy7 and ^99m^Tc-AEx-Cy7 at various concentrations (i.e., 0, 5, 10, 50 and 200 μg/mL) were added to the medium, and then the cells were incubated for another 24 h. In addition, TEx (200 μg/mL), AEx (200 μg/mL), ^99m^Tc-TEx-Cy7 (37 KBq) and ^99m^Tc-AEx-Cy7 (37 KBq) were incubated for different time points (0, 2, 6, 12,18, 24, 48 and 72 h). At the end of the incubation, CCK-8 was added and continued to incubate for 2 h. The absorbance values of the cells were measured with a microplate reader (iMarkTM Microplate Absorbance Reader, Bio-Rad, USA) at 450 nm.

### In vitro tumor cell binding

Fluorescence intensity of TEx was analyzed by flow cytometry to determine the level of internalization. The modification of exosomes was adapted from a literature reported method [28]. Briefly, we introduced 1 mg DSPE-PEG per 1 mg exosomes. TEx were incubated with 1 mg DSPE-PEG_2000_-FITC (Ruixi, Xi’an, China) at room temperature for 30 min, and FITC-TEx were passed through a centrifugal filter device (100 kDa molecular weight, Amicon®Ultra-15) as previously described [28]. For internalization assay, HCT116 cells were seeded in 10 cm cell culture dishes and treated with different concentrations of FITC-TEx (0, 5, 10, 20 μg/mL). After incubation at 37℃ for 12 h, cells were digested and dissolved in 200 µL PBS for flow cytometry analysis (FACSort, BD, USA). The tumor binding ability of TEx was assessed by confocal microscopy. TEx and DSPE-PEG_2000_-Cy5 (Ruixi, Xi’an, China) were incubated at room temperature for 30 min, and Cy5-labeled TEx (TEx-Cy5) were obtained. TEx-Cy5 (100 μg /mL) were added onto HCT116 cells grown in a confocal dish and incubated at 37℃ for different time periods (6 h, 12 h, 24 h, 48 h). The cell nuclei were counterstained with 4′,6-Diamidino-2-phenylindole (DAPI) (Boster, Wuhan, China). Cells were fixed with paraformaldehyde and observed under a Fluorescence microscope. Then TEx-Cy5 (100 μg /mL) were added onto HCT116 cells grown in a confocal dish and incubated for 12 h. The cytoskeleton of tumor cells was stained with FITC-phalloidin, and cell nuclei were counterstained with DAPI (Boster, Wuhan, China). Cells were fixed with paraformaldehyde and observed under a confocal microscope (LSM 880, ZEISS).

### TEx and AEx modification

0.5 mg of DSPE-PEG_2000_-HYNIC (Ruixi, Xi’an, China) and 0.5 mg of DSPE-PEG_2000_-Cy7 (Ruixi, Xi’an, China) were incubated with TEx (1 mg) or AEx(1 mg) for 30 min at room temperature to form HYNIC (Cy7)-PEG_2000_-DSPE-TEx (HYNIC-TEx-Cy7) and HYNIC (Cy7)-PEG_2000_-DSPE-AEx (HYNIC-AEx-Cy7), as previously described [28]. The samples were passed through a centrifugal filter device (100 kDa molecular weight, Amicon®Ultra-15) before further use.

### Radiolabeling, purification, and identification

HYNIC-TEx-Cy7/HYNIC-AEx-Cy7 (5 mg/mL, 1 mL) were incubated with 1 mL of tricine/EDDA solution (20 mg/mL tricine, 10 mg/mL EDDA at pH 6–7; Sigma/Aldrich, St. Louis, Mo, USA). The ^99^Mo/^99m^Tc generator (Beijing Atom High Tech, Being, China) was used to obtain a 740 MBq ^99m^TcO_4_^−^ solution, and 20 μL of SnCl_2_·2H_2_O (1 mg/mL in 0.1 N HCl; Sigma/Aldrich) was added. After a 30-min incubation, exosomes were purified using PD10 Sephadex G-25 (GE, USA). Radio thin-layer chromatography (radio-TLC) was used to determine the radiochemical purity with silica gel paper strips (Gelman Sciences, Germany) as a stationary phase and saline as a mobile phase. The stability of exosomes in FBS for 6 h at 37 °C was analyzed using radio-TLC. FT-IR was used to verify whether the DSPE-PEG_2000_ materials were inserted onto the exosomes.

### Cellular uptake of ^99m^Tc-TEx-Cy7

To assess cell uptake, we incubated 1 × 10^6^ of HCT116 cells with RPMI-1640 medium supplemented with 10% fetal bovine serum containing ^99m^Tc-labeled TEx (37 kBq/well) at 37 °C for 0.5, 1, 2, 3, 6, 12, 24, 36 and 48 h. HCT116 cells incubated with ^99m^Tc-labeled AEx were used as a control. After incubation, the supernatants were removed and washed with cold PBS. The remaining cells were lysed in 0.1 M NaOH and rinsed with cold PBS. Cell lysates and supernatants were collected. Radioactivity was measured using a γ-counter (PerkinElmer, USA), and the cellular uptake of ^99m^Tc-TEx-Cy7 was calculated as the radioactivity in the cells divided by the total added radioactivity and multiplied with 100 to get the percentage. Experimental conditions were performed in triplicate.

### Tumor-bearing nude mouse models

All mouse experimental procedures were reviewed and approved by the Animal Care Committee of Tongji Medical College, Huazhong University of Science and Technology. HCT116 cells (5 × 10^6^) suspended in 100 μL PBS were subcutaneously injected into the upper right leg of BALB/C nude mice (male, 4 weeks old; Beijing HFK Bioscience co., Ltd, China). After the tumor volume reached approximately 50 mm^3^, tumor-bearing mice were used for imaging.

### NIRF imaging

^99m^Tc-TEx-Cy7 were injected into tumor-bearing mice (n = 3 per group) via the tail vein for NIRF imaging. Mice were anesthetized with 2% isoflurane, and NIRF imaging was performed at different time points (1, 6, 12, 18, and 24 h after injection). Static NIRF images were acquired with 750 nm excitation and 790 nm emission filters using an IVIS Spectrum imaging system (In-Vivo FX PRO, Bruker, Germany). NIRF images were analyzed using Bruker MI (Bruker, Germany).

### SPECT imaging

SPECT imaging was performed using a SPECT MPR (GE, USA) with a 3.0-mm pinhole collimator. Briefly, after intravenous injection of 29.6 MBq ^99m^Tc-TEx-Cy7, tumor-bearing mice were anesthetized with 2% isoflurane. Images were acquired 6, 12, 18, and 24 h after the injection of ^99m^Tc-TEx-Cy7. ^99m^Tc-AEx-Cy7-injected tumor-bearing mice served as a control.

### Biodistribution analysis

To determine the metabolic characteristics of TEx, we assessed the biodistribution of ^99m^Tc-TEx-Cy7 in HCT116 tumors. HCT116 tumor-bearing mice were injected with 29.6 MBq ^99m^Tc-TEx-Cy7. Animals were sacrificed 6, 12, 18, and 24 h after injection (n = 3 mice per time point). Tissues were excised, weighed, and analyzed using a γ-counter. The radioactivity in organs and tissues was calculated as the percentage of injected dose per gram of tissue (% ID/g) and corrected for radioactive decay.

### In vivo toxicity studies

BALB/c mice (n = 5 per group) received an i.v. injection of 200 μL of PBS, or ^99m^Tc-TEx-Cy7 (37 MBq), or ^99m^Tc-TEx-Cy7 (37 MBq). On day 1st and day 7^th^ after the injection, mice were euthanized, their blood samples and major organs (i.e., hearts, livers, spleens, lungs and kidneys) were collected. A blood biochemical autoanalyzer (Chemray 240, Rayto Life and Analytical Sciences Co., Ltd, China) was applied to measure the function of liver and kidney, such as alanine amino transferase (ALT), aspartate aminotransferase (AST), and alkaline phosphatase (ALP), blood urea nitrogen (BUN), and creatinine (CRE). Hematoxylin and eosin (H&E) of major organs were examined using an optical microscope (IX73, Olympus, Japan).

### Statistical analysis

Data are shown as the mean ± standard deviation (SD). Comparisons between groups were evaluated with the unpaired Student’s t-test. *p* < 0.05 was considered to be statistically significant. Statistical analysis was conducted using GraphPad Prism v8.0 software.

## Supplementary Information

**Additional file 1: Figure S1.** FT-IR combinational spectrograms of ^99m^Tc-TEx-Cy7, TEx, DSPE-PEG_2000_-HYNIC, DSPE-PEG_2000_-Cy7.

## Data Availability

Not applicable.

## Abbreviations

Ex: Exosomes
TEx: Tumor cell-derived exosomes
ASCs: Adipose stem cells
AEx: Exosomes from adipose stem cells
SPECT: Single-photon emission computed tomography
PET/CT: Positron emission tomography/computed tomography
MR: Magnetic resonance
NIRF: Near-infrared fluorescence
HYNIC: Hydrazinonicotinic acid
Cy7: Cyanine7
DSPE-PEG_2000_-Cy7: Cy7-terminated 12-distearoyl-sn-glycero-3-phosphetha-mine-[(polyethyleneglycol)-2000]
TEM: Transmission electron microscopy
DLS: Dynamic light scattering
CCK8: Counting Kit-8
Cy5: Cyanine5
DSPE-PEG_2000_-Cy5: Cy5 labeled DSPE-PEG_2000_
DAPI: 4′6-Diamidino-2-phenylindole
^99m^Tc: Technetium-99
FBS: Fetal bovine serum
PBS: Phosphate buffer saline
SD: Mean ± standard deviation
T/M ratios: Tumor-to-muscle ratios
T/L ratios: Tumor-to-liver ratios
%ID/g: Injected dose per gram
