# Supplementary material for: Hydrophobic insertion-based engineering of tumor cell-derived exosomes for SPECT/NIRF imaging of colon cancer
Source: J Nanobiotechnology. 2021 Jan 6;19:7. doi: 10.1186/s12951-020-00746-8 (PMC7789573; doi:10.1186/s12951-020-00746-8)
Supplement: Supplementary file 1 — Additional file 1: Figure S1. FT-IR combinational spectrograms of 99mTc-TEx-Cy7, TEx, DSPE-PEG2000-HYNIC, DSPE-PEG2000-Cy7. [file 12951_2020_746_MOESM1_ESM.docx]

**Title page:**

**Hydrophobic insertion-based engineering of tumor cell-derived exosomes for SPECT/NIRF imaging of colon cancer**

Boping Jing*^1,2^*^†^, Yongkang Gai*^1,2^*^†^, Ruijie Qian*^1,2^*, Zhen Liu*^1,2^*, Ziyang Zhu*^1,2^*, Yu Gao*^1,2^*, Xiaoli Lan *^1,2^*^*^, Rui An*^1,2^*^*^.

Affiliations

*^1^* Department of Nuclear Medicine, Union Hospital, Tongji Medical College, Huazhong University of Science and Technology, Wuhan 430022, China

*^2^* Hubei Key Laboratory of Molecular Imaging, Wuhan 430022, China

^†^ Boping Jing and Yongkang Gai contributed equally to this work.

^*^ Corresponding Authors: Rui An, No. 1277 Jiefang Ave, Wuhan, Hubei Province 430022, China. Phone: +86-13986113240; Fax: +86-27-87543437. E-mail: [1975xh0577@hust.edu.cn](mailto:1975xh0577@hust.edu.cn)

Xiaoli Lan, No. 1277 Jiefang Ave, Wuhan, Hubei Province 430022, China. Phone: +86-13886193262; Fax: +86-27-85726282. E-mail: [LXL730724@hotmail.com](mailto:LXL730724@hotmail.com);


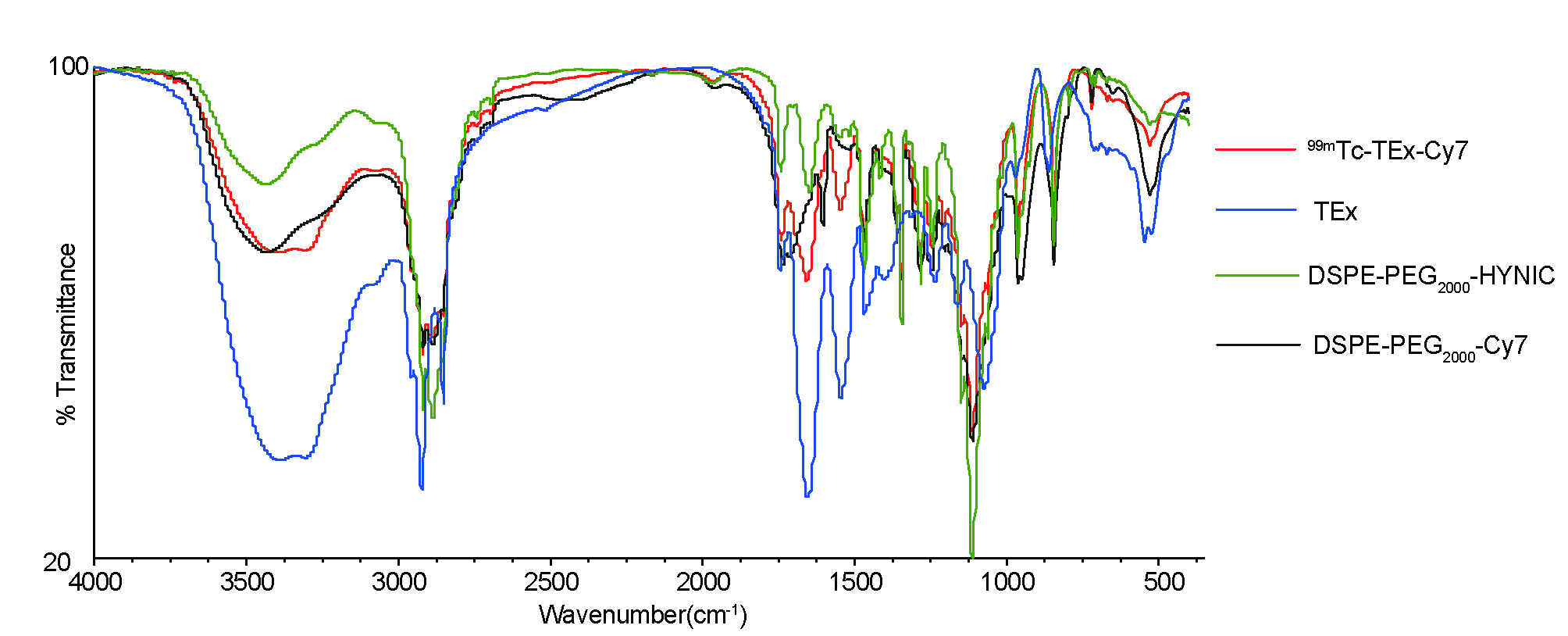
Figure S1. FT-IR combinational spectrograms of ^99m^Tc-TEx-Cy7, TEx, DSPE-PEG_2000_-HYNIC, DSPE-PEG_2000_-Cy7.
